# Large Pore Ion and Metabolite-Permeable Channel Regulation of Postnatal Ventricular Zone Neural Stem and Progenitor Cells: Interplay between Aquaporins, Connexins, and Pannexins?

**DOI:** 10.1155/2012/454180

**Published:** 2012-06-13

**Authors:** Leigh E. Wicki-Stordeur, Leigh Anne Swayne

**Affiliations:** ^1^Division of Medical Sciences, Island Medical Program, University of Victoria, Victoria, BC, Canada V8W 2Y2; ^2^Department of Biology, University of Victoria, Victoria, BC, Canada V8W 3N5; ^3^Department of Biochemistry and Microbiology, University of Victoria, Victoria, BC, Canada V8W 3P6; ^4^Department of Cellular and Physiological Sciences, University of British Columbia, Vancouver, BC, Canada V6T 1Z3

## Abstract

The birth of new neurons from unspecialized neural stem and progenitor cells surrounding the lateral ventricles occurs throughout postnatal life. This process, termed neurogenesis, is complex and multistepped, encompassing several types of cellular behaviours, such as proliferation, differentiation, and migration. These behaviours are influenced by numerous factors present in the unique, permissive microenvironment. A major cellular mechanism for sensing the plethora of environmental cues directing this process is the presence of different channel forming proteins spanning the plasma membrane. So-called large pore membrane channels, which are selective for the passage of specific types of small molecules and ions, are emerging as an important subgroup of channel proteins. Here, we focus on the roles of three such large pore channels, aquaporin 4, connexin 43, and pannexin 1. We highlight both their independent functions as well as the accumulating evidence for crosstalk between them.

## 1. Introduction

New neurons are produced in the ventricular zone (VZ) of the lateral ventricles throughout postnatal life [1]. This is a remarkable developmental process, in which unspecialized neural stem and progenitor cells (NSC/NPCs) pass through a complex gauntlet of cell behaviours, such as proliferation, differentiation, and migration. It is now becoming increasingly clear that the highly controlled movement of several ions and small molecules trigger numerous, complex signaling pathways that underscore the regulation of these behaviours (recently reviewed in [2, 3]). As follows, there is a growing body of evidence implicating “large pore” channels in the control of postnatal VZ neurogenesis. In contrast to typical ion channels, which are selective for small ions, large pore channels can additionally (or exclusively) allow passage of small molecules (neutral or charged). Aquaporin 4 (AQP4) connexin 43 (Cx43), and pannexin 1 (Panx1) are three such large pore channels that are expressed in postnatal VZ. Perhaps not surprisingly the roles of these channels appear to be closely linked with one another and also with the functions of other ion channels in the regulation of postnatal VZ NSC/NPC biology.

## 2. AQP4

There are thirteen known types of AQPs in mammals (AQP0-12; recently reviewed in [4]). These are categorized into two primary subgroups based on function: those selective solely for water (AQP0, AQP1, AQP2, AQP4, AQP5), and those permeable to water as well as small nonpolar solutes such as glycerol and urea (AQP3, AQP7, AQP9, and AQP10). Additional types can conduct ions (AQP6, AQP8), while so-called “unorthodox” members (AQP11, AQP12) are more distantly related to the other aquaporins and are expressed on intracellular membranes [5]. In general, AQP proteins are comprised of about 300 amino acids with six transmembrane *α*-helices arranged in a right-handed bundle with intracellular N- and the C-termini [6, 7]. AQP monomers oligomerize to form tetramers, generating four aqueous pores [8, 9]. Specific motifs within the interhelical loop regions form the water conduit and selectivity filter [10]. Slight variations in peptide sequence between different AQPs have generated variability in the size of the pore. This is part of the basis for water selectivity (small pore) versus simultaneous water and nonpolar solute permeability (larger pore) [8].

AQPs 1, 4, and 9 are present in the central nervous system (CNS), largely in epithelial cells, ependymal cells, and/or astroglia ([11–14], reviewed in [15, 16]), where they facilitate movement of water between blood and brain, and between brain and cerebrospinal fluid compartments. Dysregulation of cell volume in the brain underlies clinical conditions such as edema and hypoxia. Water balance also plays a crucial role in neurogenesis, as NSC/NPCs must move considerable amounts of water into or out of the cell to rapidly change their volume during proliferation, differentiation, and migration.

The major AQP found in brain, AQP4, is highly enriched in the neurogenic regions [11, 14, 17], particularly the VZ, and is the main isoform expressed in adult NSC/NPCs and ependymal cells [17, 18]. As described above, AQP4 is a member of the water-only permeable subgroup. Considerable AQP8 (water plus small nonpolar solutes) and AQP9 (water plus ions) have also been detected in NSC/NPCs in culture [18]. In contrast to AQP4, which is more ubiquitous in the VZ, AQP9 is mainly localized in NSC/NPCs in the dorsolateral corner [17]; however, its exact functional significance in NSC/NPC biology remains to be determined. AQP8 is detected primarily in the mitochondria-enriched fraction, although whether it is present in neurogenic regions *in situ* has not yet been reported [18].

Most of what is currently known about the role of AQPs in NSC/NPCs comes from recent work on AQP4 [19–21]. Using AQP4 knockout (KO) mice, Kong et al. [19] demonstrated that it controls proliferation, survival, migration, and neuronal differentiation of VZ NSC/NPCs. An observed impairment in neurosphere formation in AQP4 KO mice was attributed to both increased cell apoptosis and decreased cell proliferation due to cell cycle arrest in G2/M phase. Furthermore, upon neurosphere differentiation, the proportion of immature neurons in the AQP4 KO population was significantly lower than in the wildtype population, whereas there was no significant difference in the proportion of astrocytes. To help elucidate the underlying mechanism, the authors investigated the effects of AQP4 loss on Ca^2+^ oscillations. In NSC/NPCs, L-type Ca^2+^ channel mediated Ca^2+^ fluxes [22, 23] and purinergic receptor- (P2R-) dependent Ca^2+^ oscillations [24–27] play major roles in directing neurogenesis (recently reviewed in [2, 3]), in part through Ca^2+^-dependent transcription [23]. Interestingly, these P2R-mediated Ca^2+^ oscillations can even occur spontaneously without exogenous stimulation in NSC/NPCs [25, 26]. AQP4 KO increased the frequency but decreased the amplitude of spontaneous Ca^2+^ oscillations and suppressed high K^+^-induced Ca^2+^ influx. Given its demonstrated effects on intracellular Ca^2+^, it is not surprising that AQP4 KO also affected the expression of other channels: the expression of both Cx43 and the L-type voltage-gated Ca^2+^ channel Ca_v_1.2 subtype were reduced.

## 3. Cx43

Cxs are a family of vertebrate four-pass transmembrane proteins with intracellular N- and C-termini, that oligomerize into hexameric channels known as connexons (hemichannels), which, in turn, can connect neighboring cells across the extracellular space by formation of gap junctions [28]. These junctions provide a physical link between cells through which ions, metabolites, and other messengers of up to 1 kDa in size can diffuse, thereby mediating cell-cell communication through passage of signaling molecules such as ATP [29], IP_3_, and Ca^2+^ ([30] reviewed in [31, 32]). Gap junction-independent functions of hemichannels have also recently been identified, in which similar exchanges between the cell and its extracellular environment are facilitated (reviewed in [33]). Furthermore, the variable C-terminal domains of individual Cxs can exert intrinsic functionality independent from channel activity (reviewed in [34]), that appears to be regulated by signaling/adaptor proteins like protein kinases, phosphatases, and structural proteins (reviewed in [35]). Cxs have been shown to widely influence physiological and pathological processes and are key in coordinating metabolic and electrical activities as well as cell growth and proliferation (reviewed in [36]), cytoskeletal dynamics [37], and transcriptional regulation [38–40].

Over twenty mammalian members of the Cx family have been identified, with each respective isoform originally named for its molecular weight (reviewed in [41]). Cx43 (gap-junction protein alpha-1, Gja1) is the most widely and highly expressed Cx in almost every tissue [42], and it is the predominant isoform within the CNS. Within the developing CNS, Cx43 is detected in several cell types including astrocytes, NSC/NPCs, cortical neurons, and dopaminergic neurons of the developing midbrain [43–49]. Cx43 is critical for proper CNS formation and organization, likely through its role in the neurogenic processes of NSC/NPC proliferation [50], differentiation [47, 51], and migration [52–54] during development. Interestingly, studies in human and murine embryonic stem cells have found transcriptional regulatory elements controlled by the NSC transcription factor SOX2 within the Cx43 gene region [55] and have identified Cx43 as necessary for both neuroectodermal specification [56] and stem cell proliferation [57].

In the postnatal and adult brain, Cx43 expression becomes much more highly restricted to astrocytes [58–60]. However, Cx43 remains present in cortical neurons [61], ependymal cells [44], NSC/NPCs, and migratory neuroblasts [62–65]. Within the neurogenic VZ and subsequent rostral migratory stream (RMS), a dramatic increase in Cx43 is noted between neonatal periods and adulthood [66] in the astrocytes, NSC/NPCs, and ependymal cells, all of which exhibit gap-junction-dependent coupling [63, 64, 67]. Within this stem cell environment, Cx43 is further thought to be involved in hemichannel mediated ATP uptake and release [68, 69], contributing to propagation of Ca^2+^ waves from intracellular IP_3_-dependent stores [70, 71]. This Ca^2+^ release regulates NSC/NPC cell cycle entry and thus proliferation [72]. Cx43 hemichannels are also permeable to Ca^2+^ and controlled by Ca^2+^ (for recent studies see [73, 74]).

The data on the role of Cx43 in postnatal VZ neurogenesis is somewhat conflicting, and studies have been hindered by the lethality of the full Cx43 knock-out due to severe neonatal heart defects [75]. Some lines of evidence point to a negative regulation of proliferation by Cx43. Within the subependymal layers and RMS, levels of Cx43 were inversely correlated with lower levels of DNA synthesis [66]. Intriguingly, this correlation was only mimicked in a primary cell culture model upon high levels of confluence, indicating a potential role for Cx43 in contact inhibition. Furthermore, *in vitro* studies in mouse Neuro2a neuroblastoma cells, a commonly used NPC model, demonstrated an increased doubling-time upon Cx43 overexpression under nongap junction forming conditions. Interestingly, only the C-terminal tail was required for this reduced proliferation, possibly through transcriptional regulatory mechanisms, as this domain contains a putative nuclear localization signal [76]. Additional work in Neuro2a cells identified Cx43 as a Ca^2+^-dependent regulator of cell volume [77]. Murine PC12 cells, a well-studied pheochromocytoma-derived cell model for neurite outgrowth, exhibited enhanced NGF-induced neurite outgrowth when overexpressing Cx43. Interestingly, untransfected cells within the same dish as those overexpressing Cx43 also demonstrated enhanced neuritogenesis due to Cx43 hemi-channel-mediated ATP release [78]. Similarly, using murine embryonal carcinoma P19 cell line, Cx43 (and Gjb2) inhibition resulted in decreased astrocytic and neuronal differentiation of these cells [79]. In contrast to these results pointing to a role for Cx43 in negative regulation of proliferation, other studies suggest Cx43 is a positive regulator of proliferation. In developing and early postnatal hippocampus, conditional Cx43 knockout in radial glia and astrocytes causes severe inhibition of hippocampal NSC/NPC proliferation [80]. Moreover, embryonic cortical neurospheres were dependent on Cx43 gap junctional coupling to maintain cells in proliferative state [50], but whether this is conserved in the postnatal VZ is unknown.

Still, the functional relevance of Cx43 in NSC/NPCs of the postnatal VZ *in vivo* remains to be discovered. Currently, much is assumed from the previously mentioned cell culture experiments, as well as developmental and postnatal hippocampal studies. Together, it appears a role for Cx43 may be emerging in VZ NSC/NPC self-renewal, differentiation and migration, thereby contributing to the regulation of the postnatal process of neurogenesis.

## 4. Panx1

Panx1 is part of a three-membered family of proteins with homology to the invertebrate gap junction forming innexins [81]. However, little concrete evidence exists pointing towards gap junction functions for Panxs, which are instead widely considered single-membrane channels (reviewed in [82–84]). Panx1 monomers have a predicted four-pass transmembrane sequence, with a conserved intracellular N-terminus and much longer, variable intracellular C-terminus. These monomers oligomerize into large hexameric pores [85] that may be opened by depolarization [86, 87], increased extracellular K^+^ (independent of depolarization) [88, 89], mechanical stimulation [90], NMDAR activation [91], intracellular Ca^2+^ [92], or low oxygen and glucose conditions [93, 94]. Recently, it has been demonstrated that the C-terminal domain of Panx1 is autoinhibitory, and can be removed by caspase-dependent cleavage, resulting in constitutive activation of this channel [95, 96]. Furthermore, Panx1 activation can be inhibited by dramatically increased extracellular ATP [97] or upon cytoplasmic acidification [92], as well as through mimetic peptides [98] and channel blockers [99, 100]. Once activated, the Panx1 pore may nonselectively pass ions, metabolites, and other signaling molecules up to 1 kDa in size (reviewed in [82–84]); however, recent evidence has pointed towards Panx1 as being selective for anions (e.g., Cl^−^) and anionic small molecules [101]. These channels are involved in several physiological and pathological processes, largely by mediating ATP release in several cell types (reviewed in [82–84]).

Panx1 is found in a wide range of rodent tissues, with an expression profile similar to that of Cx43 [100]. It is abundantly expressed in the brain [102, 103]. Importantly, this relatively newly discovered large pore channel has recently been identified in postnatal VZ NSC/NPCs and their immature neuronal progeny [27]. Using Neuro2a murine neuroblastoma cells and primary postnatal VZ neurosphere cultures, Panx1 overexpression and inhibition dramatically increased and decreased NSC/NPC proliferation, respectively. Furthermore, this regulation was partly due to the ability of Panx1 to release ATP (reviewed in [82, 83, 100, 104]), a potent signalling metabolite, which is released in sporadic bursts from NSC/NPCs [25]. Released ATP triggers intracellular Ca^2+^ mobilization via activation of P2R signaling [24–27]. Ongoing studies will likely uncover additional regulatory roles of Panx1 in neurogenesis, as well as underlying mechanisms.

## 5. Crosstalk between “Large” Pore Channels and Convergence of Signaling Mechanisms

**Figure 1:**
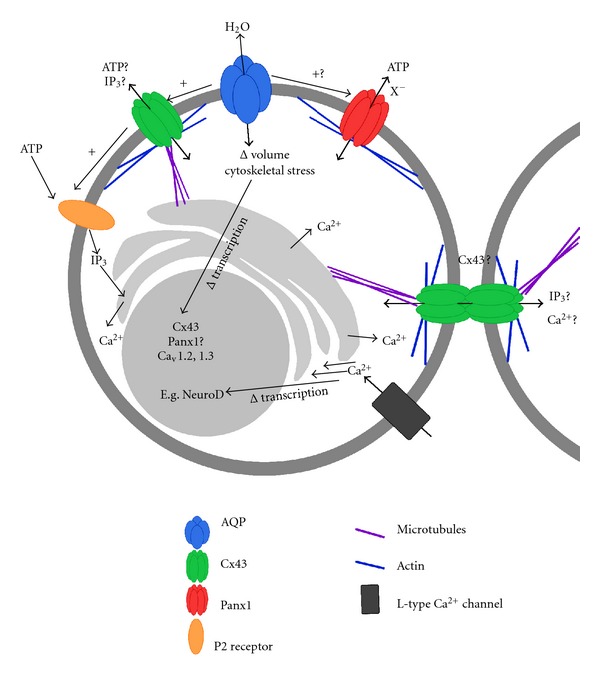
Schematic illustration of the interplay between AQP4, Cx43 and Panx1 large pore channels as they mediate cytoskeletal interactions, Ca^2+^ signaling, transcriptional regulation, ATP flux, and cell-cell communication between VZ NSC/NPCs.

Figure 1 summarizes the roles of AQP4, Cx43 and Panx1 in postnatal VZ NSC/NPCs. Interestingly, there appears to be multiple levels of crosstalk between each of these large pore channels. Here, we outline three primary interconnected ways in which the regulation and function of these large pore channels converge: solute gradient regulation, cytoskeletal signaling related to cell volume changes, and nucleotide signaling.

### 5.1. Gradient Regulation

The movement of ions and metabolites is often dependent on the ability to tightly control concentration gradients. These gradients cannot be generated and/or maintained without concomitant control of water volume. The mechanism underlying the effects of AQP4 loss on Ca^2+^ oscillations and changes in L-type and Cx43 channel expression have not been fully elucidated; however, it is conceivable that these changes could result, in part, from alterations in ion concentration gradients. Cx43 has also been implicated in volume control (for review see [105]), perhaps through reciprocal relationships with AQP4, as described above. Thus, the ion fluxes through Cx43 and Panx1 are dependent on the capacity of AQP4 to regulate solute concentration gradients.

### 5.2. Cytoskeletal Signaling

Proliferating, differentiating and migrating NSC/NPCs and neuroblasts must make specific and substantial changes in cell volume and morphology that undoubtedly require the movement of water molecules. For example, cell proliferation required for neurosphere formation is inhibited by a hypertonic medium [106]—in glioma cells this results in sustained cell swelling following transient cell shrinkage [107, 108]. The precise details of volume-sensing signaling mechanisms triggered by AQP4-mediated water movement that are important for neurogenesis remain to be further elucidated. An early study in cultured astrocytes demonstrated that AQP4 knockdown also induced alterations of the actin cytoskeleton [109]. Therefore, AQP4-mediated changes in cell volume could directly regulate Cx43 and Panx1 signaling through stretch activation of the channels and/or the cytoskeletal-associated signaling pathways to which they are linked. Recent work has demonstrated that extracellular matrix stiffness modulates NSC behaviour [110] and that cytoskeletal-regulating Rho GTPases mediate the lineage commitment of hippocampal NSCs [111]. For many years, Cxs have been closely linked to the cytoskeleton in numerous cell types (e.g., see [37, 112–116], for reviews see [105, 117, 118] with actomyosin-mediated contractility actually inhibiting Cx43 hemichannel activity [118]).

As described above, we also now know that Panx1 regulates NSC/NPC proliferation [27] which adds another layer of complexity. Previous work has shown that these channels can be activated by mechanical stress [90]. Further suggesting the potential for positive crosstalk between Panx1 and the actin cytoskeleton in NSC/NPCs, Panx1 has been demonstrated to interact with the actin cytoskeleton [119] and drive actin remodeling [120]. Moreover, nucleotide-dependent mechanisms (e.g., ATP flux, P2R signaling) are implicated in cytoskeletal remodeling in NSC/NPCs [121]. Interestingly, recent work has demonstrated that, in addition to regulating Cx43 and the actin cytoskeleton, AQP4 knockdown reduces a maxi volume-regulated anion current of unknown molecular identity [122]. Given the discovery of the anion selectivity of Panx1 [123], it is tempting to speculate that Panx1 is the molecular basis of this enigmatic maxi volume regulated anion channel—which, incidentally, also mediates ATP release [124].

### 5.3. Nulceotide Signaling

Purinergic signaling mechanisms also further link Cx43 and Panx1, albeit somewhat controversially. Prior to the discovery of Panx1, channel-mediated ATP release was mainly attributed to Cx43 hemichannels. Interestingly, Cx43 expression also regulates P2R expression [26] in embryonic VZ NSC/NPCs. Cx43 hemichannel-mediated ATP release was heavily studied in astrocytes (e.g., see [68, 125]), however, this role has recently been challenged in favour of Panx1 [126]. Importantly, while Cx43 did not appear to form hemichannels in *Xenopus *oocytes [127], numerous studies in mammalian cells have elucidated the intricacies of Cx43 hemichannel activity (e.g., see [73, 74]). Furthermore, the cross-inhibition of Cx hemichannels, Panxs, and volume-activated ion channels by certain pharmacological tools is now well known [98, 104, 128], adding further levels of complexity as several previously identified Cx channel blockers are now known to inhibit Panx1 with equal or greater efficacy. Whether Cx43 has hemichannel activity in postnatal VZ NSC/NPCs may thus be more of an open question than was previously thought and further work is clearly needed to elucidate its role. Given that we now know that Panx1 appears to play an important role in purinergic signaling in NSC/NPCs, likely in part through mediating ATP release [27], it will be important to determine if and how Panx1 and Cx43 functionally interact in the postnatal VZ. Might there be crosstalk between Cx43 and Panx1 in ATP release and downstream purinergic signaling in the postnatal VZ? Furthermore, what is the added value of having both types of channels? Distinctions between Cx43 and Panx1 signaling may potentially lie in differences in regulation by internal and external Ca^2+^ concentrations, ion selectivity, single channel conductance, and/or involvement in separate protein complexes and signaling pathways (for reviews, see [83, 84, 117, 118, 129] and also see recent developments [73]). These and other similar questions will undoubtedly be the focus of future work.

## 6. Conclusions and Perspectives

Here, we have reviewed literature on the roles of three large pore ion channels, AQP4, Cx43, and Panx1 in the regulation of postnatal VZ neurogenesis. A common thread that has emerged during this process is that the regulation and functions of these channels seem to intimately connected (Figure 1).
